# A guide to reactive oxygen species in tumour hypoxia: measurement and therapeutic implications

**DOI:** 10.1002/1878-0261.70151

**Published:** 2025-10-29

**Authors:** Lina Hacker, Elysia Sarsam, Stuart J. Conway, Ester M. Hammond

**Affiliations:** ^1^ Department of Oncology The University of Oxford UK; ^2^ Department of Chemistry & Biochemistry University of California Los Angeles California USA

**Keywords:** cancer, hypoxia, reactive oxygen species, tumour microenvironment

## Abstract

Reactive oxygen species (ROS) are a diverse group of molecules that serve as both essential signalling mediators and potential drivers of oxidative stress. In tumours, ROS influence critical processes such as proliferation, angiogenesis, metabolic adaptation and therapy resistance. These processes are further modulated by reduced oxygen availability (hypoxia), a defining feature of many solid tumours that can alter redox balance and cellular signalling. The interplay between ROS and hypoxia is highly dynamic, with both factors shaping tumour behaviour in complex and often unpredictable ways. Accurately measuring ROS and tumour oxygenation remains a significant challenge due to their transient nature and variability in levels across different tumour types. In this guide, we provide a comprehensive update on the dynamic interaction between ROS and hypoxia in tumours, evaluate current strategies for ROS detection and discuss emerging therapeutic approaches that target redox vulnerabilities in cancer. Understanding the intricate relationship between ROS and hypoxia is crucial for refining therapeutic strategies and improving patient outcomes.

## Graphical abstract

Hypoxia reshapes tumour redox landscapes by altering compartmental ROS production (mitochondria, NOX, ER, peroxisomes). Accurate interpretation requires oxygen‐contextualised measurement (live biosensors, chemical probes, EPR, LC–MS) and awareness of artefacts (reoxygenation, probe specificity). Therapeutic strategies either exploit elevated ROS to induce cell death or target antioxidant adaptations, with efficacy critically dependent on oxygenation and temporal dynamics. Created in BioRender. Hammond, E. (2025). https://BioRender.com/w3i8lv9.

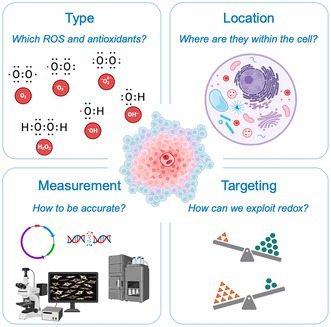

**Abbreviations**
8OHdG: oxo‐7,8‐dihydro‐2′‐deoxyguanosine
BODIPY: boron‐dipyrromethene (fluorescent dye)
CO_2_
: Carbon dioxide
DCFH: 2',7'‐dichlorodihydrofluorescein
DHE: dihydroethidium
ELISA: enzyme‐linked immunosorbent assay
EPR: electron paramagnetic resonance
ER: endoplasmic reticulum
ETC: electron transport chain
F2‐IsoPs: F2‐isoprostanes
FTC: fluorescein‐5‐thiosemicarbazide
H_2_O_2_
: hydrogen peroxide
HIF: hypoxia‐inducible factor
HNE: hydroxynonenal
HOCl: hypochlorous acid
HRP: horseradish peroxidase
LC–MS: liquid chromatography–mass spectrometry
MDA: malondialdehyde
NAC: *N*‐acetylcysteine
NADPH: nicotinamide adenine dinucleotide phosphate
NO: nitric oxide
NOX: NADPH oxidases
NRF2: nuclear factor erythroid 2‐related factor 2
O_2_
: oxygen
PnA: parinaric acid
PHD: prolyl hydroxylase
RNS: reactive nitrogen species
ROS: reactive oxygen species
SCN: thiocyanate
SOD: superoxide dismutase
TBARS: thiobarbituric acid‐reactive substances
TME: tumour microenvironment

## 1 Introduction

Tissue oxygenation is often heterogeneous, and in solid tumours, this leads to the formation of hypoxic regions driven by rapid cellular proliferation and aberrant angiogenesis [1, 2, 3]. Hypoxia profoundly alters cellular physiology, including key signalling and metabolic pathways, and disrupts redox homeostasis—the balance between oxidising and reducing species—resulting in impaired cellular function and tissue damage [4]. Redox reactions, which involve the transfer of electrons or reducing agents between biomolecules, frequently generate reactive oxygen species (ROS), a group of highly reactive molecules that act as both second messengers and mediators of cellular damage [5].

The interplay between hypoxia and ROS is complex and remains incompletely understood, partly due to inconsistent terminology, methodological limitations and the intrinsic challenges of studying transient species with low steady‐state concentrations and short half‐lives [6, 7, 8]. These factors have contributed to conflicting studies and hindered a clear understanding of redox biology in hypoxic contexts. Clarifying how ROS are generated, regulated and interpreted under low oxygen (O_2_) conditions—both in the context of signalling and detection—is critical, as hypoxia‐induced ROS influence a wide array of biological processes, including signal transduction, metabolism, cellular stress and therapy response [9, 10].

Here, we examine redox regulation in hypoxic environments, with a focus on solid tumours. We discuss the complex interplay of ROS and hypoxia, evaluate current techniques for detecting and quantifying ROS and explore therapeutic strategies that aim to target or exploit redox imbalances in cancer.

## 2 What are types, sources and location of ROS?

ROS encompasses derivatives of molecular O_2_ with different properties and reactivities, which require precise definition (Table 1) [6, 7]. They include both radical [e.g. superoxide radical anion (O_2_^•−^)] and non‐radical derivatives of O_2_ [e.g. H_2_O_2_, hypochlorous acid (HOCl) and peroxynitrite/peroxynitrous acid (ONOO^−^/ONOOH)]. The importance of specific categories of ROS in biological processes varies, with different ROS being characterised by diverse reactivities, lifespans and diffusion abilities (Table 1). For example, peroxyl radicals and H_2_O_2_ are relatively stable molecules (with half‐lives of seconds to minutes), while hydroxyl radicals are more reactive (having a half‐life of less than a nanosecond) [12]. Specific ROS can target and modify the activity, function and localisation of diverse proteins in a strictly regulated and reversible manner [13, 14, 15, 16], thereby regulating the activity of metabolic enzymes and transcription factors, as well as gene expression and epigenetic modifications.

**Table 1:** Reactive oxygen and nitrogen species (ROS/RNS) properties and reactions [7, 11].

	Chemical formula	Reactivity (rate constants, m ^−1^·s^−1^)	Lifespan	Major biological reactions
Superoxide radical anion	O_2_^•−^	Reacts with NO^•^: >10^9^ Reduces metal ions: 10^2^–10^6^	~1–10 μs (pH‐dependent)	Forms peroxynitrite (ONOO^−^) with NO^•^ Reduces Fe^3+^ and Cu^2+^, reaction rate depends on metal ligand Can damage Fe–S cluster enzymes
Hydrogen peroxide	H_2_O_2_	Reacts with thiols: 1–10 Fenton reaction with Fe^2+^: 10^2^–10^7^	Minutes to hours	Oxidises thiols and metal ions Forms ^•^OH via Fenton chemistry Reacts with CO_2_ to form peroxymonocarbonate (HCO_4_^−^)
Hydroxyl radical	^•^OH	Diffusion‐limited reactions: 10^9^–10^10^	~1 ns (10^−9^ s)	Reacts non‐selectively with DNA, lipids, and proteins
Peroxynitrite	ONOO^−^/ONOOH	Reacts with thiols and metal centres: 10^6^–10^7^ Reacts with CO_2_: 10^4^–10^5^	~1 ms (pH‐dependent)	Forms ^•^OH and NO_2_^•^ upon homolysis Reacts with CO_2_ to form ONOOCO_2_^−^
Carbonate radical anion	CO_3_^•−^	Oxidises guanine, cysteine, tyrosine, tryptophan: 10^6^–10^8^	~1 μs	Formed from CO_2_ + peroxynitrite or reaction with ^•^OH
Hypohalous acids	HOCl, HOBr	Reacts with thiols and methionine: 10^6^–10^8^ Reacts with amines: 10^4^–10^5^	Seconds to minutes	Generates chloramines/bromamines Reacts with SCN^−^ to form HOSCN, specific for thiols
Singlet oxygen	^1^O_2_	Reacts with biomolecules: 10^5^–10^7^	~4 μs (water) ~100 μs (lipids)	Produced via photosensitisation or peroxyl radical reactions
Nitrogen dioxide radical	NO_2_^•^	Oxidises thiols and lipids: 10^6^–10^7^	~1 s	Generated from peroxynitrite or NO_2_^−^ oxidation Forms nitrated biomolecules (e.g., 3‐nitrotyrosine)

ROS are scavenged by antioxidants, a general term for enzymes and small molecules that reduce oxidative damage and modulate redox signalling [6]. Each antioxidant has specific chemistry and reactivity with different ROS. Major *in vivo* antioxidants are enzymatic systems such as superoxide dismutase (SOD) for O_2_^•−^ and peroxidases for H_2_O_2_, while many low‐molecular‐mass compounds used as ‘antioxidants’ are actually scavengers of certain ROS with limited reactivity [7]. For instance, *N*‐acetylcysteine (NAC) has modes of action beyond ROS scavenging, such as increasing cellular cysteine and enhancing glutathione levels, modulating protein thiol status and generating hydrogen sulfide, which support intracellular antioxidant capacity and influence multiple redox‐dependent processes, making it difficult to attribute its effects solely to direct ROS scavenging [17]. Thus, the broader impact of such compounds often reflects redox modulation rather than direct antioxidant activity.

To attribute an effect to an antioxidant, the specific chemical species targeted should be identified, and the antioxidant effect should be chemically plausible based on specificity, rate constant, location and concentration within the cell [7]. This requires consideration of factors such as reaction kinetics (rate constants), molecular specificity, subcellular localisation and intracellular concentration. For example, attributing the scavenging of superoxide radicals (O_2_^•−^) to mitochondrial SOD is chemically plausible, as the enzyme is specifically localised in the mitochondrial matrix, has a high rate constant for reacting with superoxide and is present at sufficient concentrations to effectively outcompete other potential reactions [18]. However, accurately assessing these parameters remains challenging due to the transient nature of ROS, their compartmentalised production and the limited availability of tools that can measure specific ROS species in live cells with sufficient resolution. Moreover, measuring molecules present at low concentrations, such as ROS, often perturbs their native levels and may unintentionally alter the system under study. As such, interpreting antioxidant effects requires cautious experimental design and, ideally, converging lines of evidence. Ultimately, the ratio between ROS‐generating and ROS‐eliminating enzymes lies in a careful balance that defines the mode of downstream redox events.

**Fig. 1:**
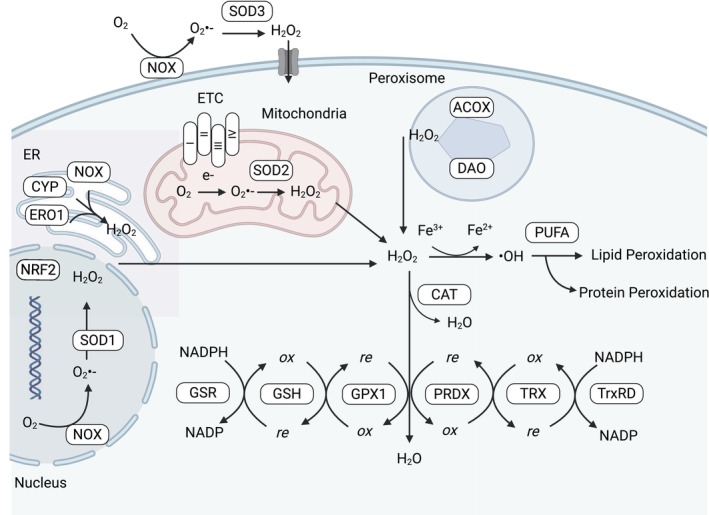
Intracellular sources and regulation of ROS. Schematic illustration of the major cellular compartments involved in ROS production and detoxification. Mitochondria produce superoxide (O_2_^•−^) primarily via the electron transport chain (ETC), which is rapidly dismutated to hydrogen peroxide (H_2_O_2_) by mitochondrial superoxide dismutase (SOD2). The endoplasmic reticulum (ER) contributes to ROS generation through ER oxidoreductin 1 (ERO1), cytochrome P450 (CYP) enzymes and NADPH oxidases (NOX), producing H_2_O_2_ as a by‐product. NOX enzymes also generate O_2_^•−^ at the plasma membrane and in the nucleus. Peroxisomes contribute via enzymes such as acyl‐CoA oxidase (ACOX) and d‐amino acid oxidase (DAO), which generate H_2_O_2_. Extracellular superoxide is converted to H_2_O_2_ by extracellular SOD3. ROS detoxification occurs through: Catalase (CAT) converting H_2_O_2_ to water, Glutathione (GSH) system involving glutathione peroxidase (GPX1), glutathione reductase (GSR), Thioredoxin (TRX) system involving peroxiredoxins (PRDX) and thioredoxin reductase (TrxRD), all dependent on NADPH to maintain reducing capacity. If H_2_O_2_ is not neutralised, it can react with Fe^2+^ via Fenton chemistry to form hydroxyl radicals (•OH), leading to lipid and protein peroxidation. The transcription factor NRF2 is activated in response to ROS and upregulates antioxidant genes to restore redox homeostasis. Created with Biorender.

The site of ROS formation plays an important role in determining cellular effects [5]. Cellular ROS derive from both enzymatic and non‐enzymatic processes (Fig. 1). Mitochondria are the primary site of ROS production, where the electron transport chain can leak electrons, forming O_2_^•−^ [19]. Peroxisomes produce H_2_O_2_ through fatty acid oxidation [20], and endoplasmic reticulum‐associated enzymes can also contribute to ROS generation during protein folding, which can reach the cytoplasm via channels such as the aquaporins [21]. NADPH oxidases (NOX) are another significant source, producing superoxide and H_2_O_2_ as part of immune responses and signalling [22]. In humans, over 40 ROS‐generating enzymes have been identified [21]. Notably, ROS levels also appear to be sex specific. In females, mitochondria produce lower levels of ROS compared to males, accompanied by reduced levels of antioxidant enzymes, which are attributed to the action of oestrogens [23]. These diverse sources highlight the complex regulation and roles of ROS in cellular physiology and pathology. Further details on the roles of the individual cellular compartments in ROS formation are thoroughly reviewed elsewhere [24].

## 3 Redox balance under hypoxia in cancer

**Fig. 2:**
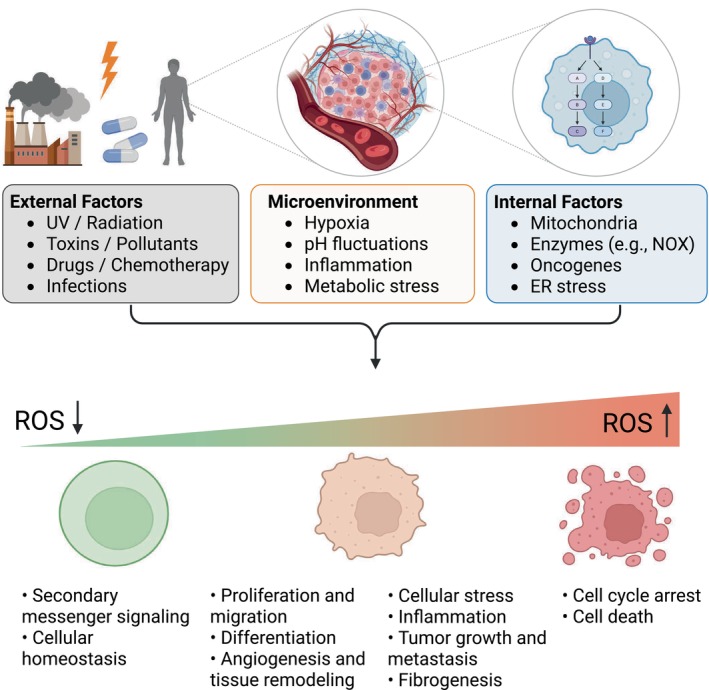
Sources and cellular consequences of elevated ROS. ROS can be generated through a range of stimuli broadly categorised as external factors (e.g. UV radiation, environmental toxins, chemotherapy, infections), internal factors (e.g. mitochondrial respiration, NADPH oxidase [NOX] activity, oncogene activation and endoplasmic reticulum stress), and microenvironmental conditions (e.g. hypoxia, acidosis, inflammation and metabolic stress; categories listed in random order). Together, these factors contribute to increased intracellular ROS levels. ROS exert dose‐dependent effects on cells: At low levels, ROS function as secondary messengers involved in maintaining cellular homeostasis and signalling. At moderate levels, they promote proliferation, differentiation, migration, angiogenesis and tissue remodelling. At high levels, ROS contribute to cellular stress, inflammation, tumour progression, fibrogenesis and metastasis. At excessive levels, ROS lead to cell cycle arrest and cell death. Created with Biorender.

ROS are crucial regulators of cellular processes, modifying target proteins directly through post‐translational modifications on redox‐sensitive residues, including cysteine and methionine [25] (Fig. 2). These modifications enable ROS to function as signalling molecules that influence cellular activities, which have been reviewed extensively [10, 21, 26, 27], and are therefore not the primary focus here. Briefly, at low to moderate levels, ROS participate in essential processes, including pathogen defence, cell signalling and mitogenic responses, by triggering pathways that promote cell proliferation and survival. However, when ROS levels exceed a certain threshold, they disrupt the delicate balance between pro‐oxidants and antioxidants, causing oxidative stress, a condition leading to cellular damage across DNA, lipids and proteins, which in turn impairs cell function and can result in cell death [28, 29]. These conflicting roles of ROS underscore their importance in health and disease, especially in pathological conditions like cancer, neurodegenerative disorders, cardiovascular diseases and diabetes [28].

In cancer cells, ROS levels are usually elevated and maintained within a range that supports pro‐tumourigenic signalling, activating pathways that promote survival without causing cytotoxicity. The causes of increased ROS in tumours are diverse and include oncogenic signalling, metabolic reprogramming, chronic inflammation and biophysical features of the tumour microenvironment (TME). Among these, hypoxia—a hallmark of solid tumours driven by disorganised vasculature and high metabolic demand—emerges as a particularly potent and complex modulator of ROS [30]. Hypoxia can reshape the balance between ROS production and clearance, largely by altering electron transport chain dynamics and impairing antioxidant defences [31, 32, 33, 34, 35, 36, 37, 38]. Together, these interactions between hypoxia and ROS establish a complex redox landscape that supports tumour evolution and therapy resistance, highlighting the need for closer examination.

## 4 How does hypoxia impact ROS?

Hypoxia plays a central role in shaping the TME by triggering widespread cellular and systemic responses. This can occur under various O_2_ concentration thresholds, with mild hypoxia typically defined as O_2_ levels below 5% and severe hypoxia below 1% [39]. ‘Normoxia’ is almost universally used to describe the ‘normal’ O_2_ levels in tissue culture flasks, that is 18–21% O_2_ (160 mmHg). 5% O_2_ (38 mmHg) is recognised as ‘physoxia’ [40], but it is important to note that physoxia can vary between tissues, and thus, this benchmark should be applied with contextual consideration. Within hypoxic regions, O_2_ deprivation stabilises hypoxia‐inducible factors (HIFs), transcriptional regulators that drive the expression of genes promoting angiogenesis, metabolic reprogramming, immune evasion and resistance to apoptosis. Systemically, hypoxia influences the recruitment and polarisation of immune cells such as macrophages, neutrophils and T cells, often reprogramming them to tumour‐promoting phenotypes [41].

The impact of hypoxia on ROS levels remains a topic of debate. While the majority of studies suggest that hypoxia increases ROS production [31, 32, 33, 34, 35, 36, 37, 38, 42], some indicate the opposite [43, 44, 45, 46, 47]. This discrepancy likely arises from differences in experimental conditions, cell types and measurement techniques.

### 4.1 Evidence supporting increased ROS in hypoxia

Many studies show an increase of ROS levels in hypoxia [31, 32, 33, 34, 35, 36, 37, 38, 42, 48, 49] and, most of these reports investigate the role of mitochondrial ROS in regulating the hypoxic response, primarily through the stabilisation of HIF. Mitochondrial complex III is thought to be a major source of ROS during moderate hypoxia, contributing to the stabilisation of HIF‐1α, a key regulator of cellular adaptation to low O_2_. ROS generated at the complex IIIQo site lead to the formation of H_2_O_2_, which inhibits prolyl hydroxylase (PHD) enzymes, preventing HIF‐1α degradation [50, 51]. Experimental findings further support this idea: silencing of Rieske iron–sulphur protein, a component of complex III, prevents HIF‐1α stabilisation [31, 36], and redox‐sensitive RoGFP probes detect increased ROS in the cytosol and mitochondrial intermembrane space under hypoxia [52, 53]. Additionally, small molecule inhibitors such as Suppressors of site IQ Electron Leak (S1QELs) and Suppressors of site IIIQo Electron Leak (S3QELs), which specifically reduce complex III ROS, also lower HIF‐1α levels, suggesting that ROS play a role in HIF‐1α stabilisation [54, 55]. Beyond HIF‐1α regulation, hypoxia‐induced mitochondrial ROS can also activate NOX leading to further ROS increase and cellular damage [56].

### 4.2 Evidence against increased ROS in hypoxia

Other studies challenge the idea that hypoxia universally increases ROS production. It has been shown that ROS generation in isolated mitochondria is typically dependent on O_2_ availability, with lower O_2_ levels resulting in reduced ROS production [43, 44, 45, 46, 47]. It was also found that ROS production via reverse electron transfer depends linearly on O_2_ levels, while ROS production from complex I and III follows a hyperbolic trend [47]. This suggests that mitochondria alone may not generate excess ROS in hypoxia and that additional cellular factors may be required. Consistent with this, other findings report no significant ROS increase in multiple cell types, including 143B, HEK293 cells and fibroblasts under hypoxia [57, 58, 59]. Two studies found that ROS levels actually decreased in human fibroblasts and osteosarcoma cells as O_2_ levels decreased (0.5% O_2_ and 1% O_2_, respectively) [60, 61]. An absence of increased ROS levels under hypoxia was observed in primary neurons and pancreatic islet cells [62, 63]. Moreover, overexpression of antioxidant enzymes such as Mn‐SOD and glutathione peroxidase‐4 failed to alter HIF‐1α stabilisation under hypoxia in certain models [62], suggesting that while ROS may modulate HIF regulation, the oxygen‐dependent inhibition of PHDs remains the dominant mechanism for HIF stabilisation. Other studies support the idea that ROS production may not be required for HIF activation [64, 65, 66]. Antioxidants such as Trolox or NAC have been shown to block hypoxic ROS production but do not alter HIF‐1α levels, whereas inhibition of ATP synthase prevents HIF‐1α accumulation by increasing cytosolic O_2_, which enhances PHD activity and promotes HIF‐1α degradation [65]. Disruption of respiratory complexes in another study similarly impairs HIF‐2α stabilisation under hypoxia, but antioxidant treatment has no effect, suggesting a ROS‐independent mechanism [67]. It has also been suggested that low‐level ROS formation can enhance PHD activity under both normoxic and hypoxic conditions, thereby reducing HIF stabilisation [64]. These varied results highlight the complexity of the regulation, suggesting that while ROS may contribute to HIF‐α modulation under hypoxia, they are not essential, with mitochondrial oxygen distribution likely playing the dominant role. A critical evaluation of the diverse and conflicting results in ROS research under hypoxia requires considering key experimental and biological factors that contribute to the inconsistencies in the literature.

### 4.3 Considerations when studying hypoxia

#### 4.3.1 Unclear terminology and ROS localisation

There is often a lack of precise cellular ROS localisation data, as well as limited specificity in ROS types and detection methods [7], complicating efforts to define the exact contribution of different ROS sources to hypoxic responses. Standardising and improving specificity in ROS terminology, such as clearly indicating the ROS species measured and their subcellular location, is essential for resolving these discrepancies.

#### 4.3.2 Limitations of ROS detection methods

Common and accessible ROS probes, such as 2′,7′‐dichlorodihydrofluorescein (DCFH), Amplex™ Red and MitoSOX™ (see Section 5), often lack specificity and can react with other molecules including reactive sulfide species, leading to inaccurate readings [7, 68]. More precise techniques, such as genetically encoded probes or liquid chromatography–mass spectrometry (LC–MS) for dihydroethidium (DHE) oxidation products, provide more reliable data [69, 70, 71] (Section 5). To address the specificity limitations of common ROS probes, careful selection of appropriate experimental controls is equally important [17].

#### 4.3.3 Cell‐type specificity and experimental variability

Oxygen metabolism varies between cell types due to differences in oxygen‐consuming enzymes [72, 73]. Different cell types exhibit varying O_2_ consumption rates, which can affect pericellular O_2_ levels and, in turn, influence ROS production in both physoxic and hypoxic conditions [74, 75]. For example, U937 cells (non‐Hodgkin lymphoma) use oxygen at a rate ten times as high as PC‐3 cells (prostate adenocarcinoma) (4 amol·cell^−1^·s^−1^ vs. 45 amol·cell^−1^·s^−1^, respectively) [75]. Moreover, specific isoforms of respiratory chain subunits and the endogenous levels of molecular and enzymatic antioxidants differ between cell types, which affects the balance between ROS production and clearance [76, 77].

#### 4.3.4 Temporal variations

The duration of hypoxic exposure and the timing of ROS measurement are crucial in understanding ROS dynamics. A transient O_2_^•–^ burst has been observed within the first 10–20 min of hypoxia, followed by a decline, which is absent in cells lacking mitochondrial DNA (ρ_0_ cells) and those treated with an O_2_^•–^ scavenger [78]. Interestingly, pre‐exposure to ambient air delays this burst and slows its decline compared to cells transferred from physoxia to hypoxia, highlighting the impact of prior O_2_ conditions. Prolonged hypoxia may trigger adaptation through HIF‐1 target gene upregulation, influencing ROS levels over time. Some studies report sustained ROS production in cells exposed to hypoxia for a longer time frame (hours–days) [79], while others show no change or even a decrease [62]. This variability highlights the uncertainty surrounding both the magnitude and timing of ROS changes, underscoring the need to carefully account for exposure duration and experimental conditions when assessing ROS levels in hypoxic environments. Sudden changes in oxygenation, such as acute hypoxia or hypoxia‐reoxygenation, can generate ROS increases significant enough to inflict oxidative damage on multiple biomolecular targets in tumours. For example, acute (cycling) hypoxia, referring to a transient reduction in oxygen availability, has been shown to significantly increase plasma malondialdehyde (MDA) and tumour 8‐oxo‐dG (8‐oxo‐2′‐deoxyguanosine) levels in transgenic *in vivo* breast cancer models, confirming lipid peroxidation and oxidative DNA damage [80]. Distinguishing the effects of acute versus chronic hypoxia, including their duration, is therefore critical and should be carefully considered in experimental design.

#### 4.3.5 Reoxygenation artefacts

Many hypoxia studies use tri‐gas incubators or modular chambers, but in the absence of a glove box, ROS measurements often involve exposure to ambient air. Even brief re‐exposure to O_2_ after hypoxia exposure can trigger a rapid ROS increase, as noted above, leading to misleading experimental results. Superoxide production can rise within 20 s of reoxygenation [81]. To avoid this, the use of controlled hypoxic workstations is recommended, ideally in combination with fixable probes (Section 5) to preserve accurate redox states during downstream analysis.

#### 4.3.6 Non‐physiological O_2_ conditions in cell culture

Standard CO_2_ incubators expose cells to ~18% O_2_, which is significantly higher than physiological O_2_ levels (~5% O_2_). This alters ROS metabolism and HIF responses [82, 83]. Cells exposed to higher O_2_ levels exhibit increased NOX activity, leading to elevated basal H_2_O_2_ levels [84]. In these conditions, PHDs hydroxylate HIF‐1α more efficiently, promoting its degradation and limiting HIF‐1α stabilisation [85]. Additionally, pericellular O_2_ levels fluctuate depending on cell density and media volume, which can impact experimental outcomes [74, 82, 83]. Cells grown in 18% O_2_ exhibit higher basal H_2_O_2_ levels due to NOX activity [86] and show altered transcriptional and metabolic responses to hypoxia compared to those cultured at 5% O_2_ [87].

#### 4.3.7 The role of culture media composition

The composition of culture media plays a crucial role in regulating cellular metabolism and redox status [88]. Replacing classical media with physiologically relevant media has been shown to increase respiratory activity and ROS (especially mitochondrial superoxide) in several cell lines [89, 90], though some studies report decreased H_2_O_2_ in C2C12 myofibroblasts under similar conditions, possibly due to higher antioxidant enzyme activity [91].

In summary, while hypoxia has been reported to increase ROS in many contexts, the results need to be evaluated with care, as they are highly influenced by measurement methods and experimental conditions, such as cell type, duration of hypoxia, O_2_ tension and metabolic state. Rather than assuming a direct and universal relationship between hypoxia and ROS, it is more accurate to view ROS production as a context‐dependent and temporally dynamic phenomenon, in which both magnitude and direction of ROS are shaped by intrinsic cellular factors and extrinsic experimental conditions. An overall increase in total ROS levels may mask compartment‐specific decreases, or vice versa, highlighting the need for temporally and spatially resolved ROS measurement that can distinguish transient bursts from sustained shifts under varying oxygen conditions in different cellular compartments. It is also important to recognise that experimental ROS measurements reflect the net balance between ROS production and antioxidant‐mediated clearance, not just production rates. Consequently, two studies using similar oxygen tensions may report opposing results if the antioxidant capacity of the studied cells differs, or if ROS buffering systems are differentially engaged during the hypoxic response. Furthermore, most studies discussed here have been conducted *in vitro*, under conditions that do not recapitulate an *in vivo* environment. Tumours exist in a state of cyclic or intermittent hypoxia rather than continuous hypoxia [92, 93], and it has been shown that intermittent hypoxia promotes higher ROS formation compared to sustained hypoxia [94]. This not only complicates experimental interpretation but also suggests that intermittent hypoxia may exert a qualitatively different influence on ROS signalling and cellular adaptation compared to steady‐state hypoxia, reinforcing the importance of incorporating physiologically relevant oxygen fluctuations into model systems. A key step in clarifying the complex relationship between ROS and hypoxia is therefore twofold: first, the adoption of more selective and quantitative ROS detection strategies capable of capturing compartment and time‐specific changes; and second, the standardisation of experimental models that better reflect physiological oxygen gradients and tumour heterogeneity. Only through this combined methodological and conceptual refinement will it be possible to reconcile contradictory findings in the literature and to develop a more coherent understanding of how hypoxia‐driven ROS dynamics shape cellular behaviour within the TME.

## 5 What are the current methods available for measuring ROS in hypoxia?

Accurate quantification of ROS is challenging due to their short half‐lives, low steady‐state concentrations and rapid fluctuations [7], especially under hypoxic conditions, where factors such as reoxygenation, magnitude and duration of the exposure can significantly influence ROS levels. ROS can be measured (1) *directly*, for example, through the use of small‐molecule fluorescent probes or electron paramagnetic resonance (EPR) (Table 2), or (2) *indirectly* by the measurement of oxidative damage markers (Table 3) [135]. Each of these methods has distinct advantages, limitations and considerations that need to be carefully navigated to obtain reliable and meaningful results. Valuable guidelines and recommendations have been previously compiled [7].

**Table 2:** Overview of direct ROS measurement methods.

Measurement method	Description	ROS species	Sensitivity	Application	References
EPR spectroscopy	Direct detection of free radicals using spin trapping	O_2_^−^, OH, RO_2_, RO, HO_2_	High	*In vivo*, *ex vivo*, *in vitro*	[95, 96, 97, 98, 99, 100, 101, 102, 103]
2′,7′‐dichlorofluorescin diacetate (DCFH‐DA)	Fluorescent probe that reacts with ROS to form DCF, detected via fluorescence	H_2_O_2_, hydroxyl radicals	Moderate	*In vitro*	[33, 35, 104]
Dihydroethidium (DHE)	Fluorescent probe that detects superoxide radicals in tissues and cells	Superoxide	High	*Ex vivo*, *in vitro*	[37, 105, 106]
D‐ROMs Test	Colorimetric test measuring hydroperoxides (ROOH) in blood plasma via Fenton reaction, producing coloured radicals	Hydroperoxides (lipid, protein, DNA peroxidation products)	Moderate to high	*Ex vivo* (plasma, serum)	[107, 108]
Amplex Red Assay	Measures hydrogen peroxide levels by reacting with horseradish peroxidase (HRP) to form resorufin	H_2_O_2_	High	*Ex vivo*, *in vitro*	[11, 32, 109]
Boronate probes (e.g. PO1, MitoB, MitoPY1, boronate‐caged luciferin)	Detects oxidation via fluorescence or LC–MS	H_2_O_2_, ONOO^−^	High		[110]
Genetically encoded thiol‐based probes (HyPer7, roGFP2‐Orp1, roGFP2‐Tsa2)	Fluorescence‐based H_2_O_2_ detection using protein sensors	H_2_O_2_	High		[38, 111, 112, 113, 114, 115]

**Table 3:** Overview of indirect ROS measurement methods. BODIPY, Boron‐Dipyrromethene (a fluorescent dye); Cys, Cysteine; ELISA, Enzyme‐Linked Immunosorbent Assay; FTC, Fluorescein‐5‐Thiosemicarbazide; LC–MS, Liquid Chromatography–Mass Spectrometry; Met, Methionine; PnA, *cis*‐Parinaric Acid; TBARS, Thiobarbituric Acid Reactive Substances; UPLC–MS/MS, Ultra‐Performance Liquid Chromatography–Tandem Mass Spectrometry.

Biomarker type	Target molecule	Common biomarkers	Detection methods	Considerations	Reference
Lipid peroxidation	Polyunsaturated fatty acids	4‐Hydroxynonenal (HNE), Malondialdehyde (MDA), F2‐Isoprostanes (F2‐IsoPs)	LC–MS, ELISA (for F2‐IsoPs), TBARS (low specificity), BODIPY fluorescence, PnA fluorescence	LC–MS is preferred; avoid artefactual oxidation during storage	[116, 117, 118, 119, 120]
Protein damage	Amino acids in proteins	Protein carbonyls, Cysteine oxidation, Methionine sulfoxide	ELISA, LC–MS, Fluorescein‐5‐thiosemicarbazide (FTC) assay, Redox proteomics	LC–MS provides specificity; oxidation can be reversible (Cys, Met) or irreversible	[5, 121, 122, 123, 124, 125, 126, 127, 128]
Nucleic acid damage	DNA & RNA	8‐Oxo‐7,8‐dihydro‐2′‐deoxyguanosine (8OHdG), 8‐Hydroxyguanosine (8OHG)	UPLC–MS/MS, Comet assay (with DNA repair enzymes), Immunohistochemistry	ELISA lacks sensitivity: UPLC–MS/MS is best; avoid spurious oxidation	[11, 129, 130, 131, 132, 133, 134]

### 5.1 Direct detection methods

Fluorescent probes are among the most widely used methods for ROS detection in cells [136] and are frequently applied in studies of hypoxia [31, 32, 35, 37, 42, 48, 137]. 2′,7′‐Dichlorofluorescin diacetate (DCF‐DA) is widely used to detect H_2_O_2_, hydroxyl radicals (^−^OH) and peroxyl radicals (ROO^−^) [35, 104], while superoxide molecules (O_2_^−^) can be detected with DHE, which is oxidised to fluorescent ethidium bromide inside cells, leading to a measurable red fluorescence signal [37, 105, 106]. Boronate‐based probes primarily detect H_2_O_2_ through selective oxidation to phenols [110], offering high reliability but limited sensitivity at physiological concentrations. Horseradish peroxidase (HRP)‐oxidising substrates, such as Amplex Red, are also frequently used for H_2_O_2_ detection [32, 109]. One of the key advantages of fluorescent detection techniques is their ease of use and ability to perform high‐throughput screening, making them popular in cell‐based assays. However, particularly in hypoxic assays, these probes face several challenges: their fluorescence signals can be directly influenced by low O_2_ levels, altered cellular pH, probe concentration and the presence of antioxidants or metal ions, all of which can lead to misinterpretation. Some of these probes, such as DCFH, also lack specificity [7, 109]. Artefacts can arise from probe accumulation in specific cellular compartments or the intercalation of oxidation products into DNA, which can influence fluorescence readings [138]. Therefore, the use of fluorescent probes requires careful controls to ensure that the observed fluorescence is due to the targeted ROS species, and thorough oxygen‐level calibration for hypoxia experiments.

In recent years, genetically encoded fluorescent sensors have emerged as powerful tools for ROS measurement [111, 112, 113, 139, 140, 141] and have helped to gain major insights in hypoxia‐related ROS research [38, 114, 115]. These sensors usually contain a dithiol switch that changes the overall fluorescence of the probe depending on its oxidation status, thereby providing high specificity and sensitivity for particular ROS species and allowing for real‐time monitoring in live cells. Genetic tools are advantageous due to their ratiometric nature, ensuring output consistency regardless of probe expression. However, probe expression can disrupt cellular function, affect ROS production and alter redox balance. Unlike traditional chemical ROS probes that can often be fixed and imaged post‐treatment, genetically encoded sensors typically need to be imaged live because they report dynamic and reversible redox changes that are lost upon fixation. In hypoxic studies, this may necessitate specialised equipment such as stage‐top incubators with controlled gas environments. Sensitivity limitations and complex calibration, requiring reducing agents, are additional challenges.

EPR (or ESR, electron spin resonance) represents an alternative direct detection technique of ROS, particularly for those with unpaired electrons, such as superoxide (O_2_^•−^) [7, 142]. EPR identifies ROS with high specificity by detecting their characteristic signals, offering the advantage of direct detection without chemical trapping, even at low concentrations. While the technique has been used to study hypoxia‐induced ROS [95, 96, 97], EPR typically requires relatively large sample volumes (live or flash frozen) and sophisticated instrumentation, limiting its application in living cells or tissues where ROS concentrations are transient and highly dynamic and may not be able to detect the full range of ROS species present in biological contexts [11]. Additionally, EPR often requires the use of spin‐trapping agents to stabilise reactive intermediates, which can complicate the interpretation of data, especially in hypoxic studies, as their reactivity could differ under low‐oxygen conditions.

### 5.2 Indirect detection methods

The short‐lived nature of ROS makes direct detection with high accuracy and precision challenging, especially in living subjects. An alternative approach is measuring oxidative damage biomarkers, which reflect the balance between ROS generation and repair or removal processes (Table 3). Oxidative damage occurs when ROS attack cellular macromolecules such as lipids, proteins and nucleic acids, leading to quantifiable modifications. Given the complexity and variability of ROS dynamics, careful experimental design and a thorough understanding of the underlying chemical processes are essential. Particularly in hypoxic systems, damage markers can be confounded by changes in repair enzyme activity, altered metabolic turnover, or hypoxia‐induced adaptive mechanisms, requiring cautious interpretation.

Lipid peroxidation occurs when ROS oxidise polyunsaturated fatty acids, generating various end products such as 4‐hydroxynonenal (HNE), MDA and F2‐isoprostanes (F2‐IsoPs) [143, 144]. These can be detected using LC–MS, ELISA (for F2‐IsoPs), TBARS (though with low specificity), and fluorescence‐based assays like BODIPY or *cis*‐parinaric acid (PnA) [116, 117, 118, 119]. Protein oxidation results in modifications such as protein carbonyls, cysteine oxidation, and methionine sulfoxide [121, 122, 123, 124, 125], which can be analysed by ELISA, LC–MS, fluorescein‐5‐thiosemicarbazide (FTC) assays, or redox proteomics [126, 127]. While some oxidative modifications are reversible (e.g. cysteine and methionine oxidation), others are permanent [5, 125, 128]. Nucleic acid oxidation, particularly guanine oxidation to 8‐oxo‐7,8‐dihydro‐2′‐deoxyguanosine (8OHdG) and 8‐hydroxyguanosine (8OHG), is commonly assessed using UPLC–MS/MS, the comet assay (enhanced by DNA repair enzymes), or immunohistochemistry [11, 129, 130, 131]. However, ELISA‐based nucleic acid oxidation assays often lack specificity and should be used cautiously [132, 133]. Across all biomarker measurements, careful sample handling is essential to prevent artefactual oxidation, and LC–MS‐based methods remain the gold standard for specificity, sensitivity and quantification [145].

Oxidative damage biomarkers are advantageous as they capture the long‐term effects of ROS on cellular integrity, offering valuable insights into the biological consequences of oxidative stress. Notably, oxidative damage arising from hypoxia‐induced ROS has been observed primarily during fluctuations in oxygen levels, such as acute or intermittent hypoxia [80, 146, 147, 148, 149]. In contrast, chronic hypoxic conditions tend to elicit a more subdued oxidative response. For example, it has been shown in several studies that 8‐oxo‐dG did not differ significantly between chronic (<0.1% O_2_ up to 24 h/0.2% O_2_ for 72 h) and normoxic conditions [48, 150], but chronic hypoxia can induce transcriptional stress through R‐loop accumulation [48]. It should be considered that the measurement of oxidative damage biomarkers is influenced not only by ROS production but also by the efficiency of cellular repair mechanisms, the removal of damaged molecules, and the process of biomarker degradation [129]. Additionally, the isolation of biomarkers during sample preparation can introduce artefacts, potentially leading to overestimation or underestimation of the extent of oxidative damage. This makes it essential to consider the biological context and the repair processes when interpreting oxidative damage data.

### 5.3 *In vivo* measurement approaches

Notably, due to the short lifespan of ROS and the risk of reoxygenation artefacts, *ex vivo* measurements should be avoided, and assessments should ideally be conducted in living cells or tissues. However, *in vivo* approaches remain limited as they require highly sensitive, non‐invasive techniques that are able to capture rapid and spatially confined signals without disturbing the physiological environment, while also overcoming barriers such as tissue penetration, motion artefacts and immune responses. While genetically encoded redox biosensors provide high accuracy and precision, their use is restricted to preclinical research settings. Injectable fluorescent or bioluminescent probes have also shown promise in animal studies [151], although their clinical application is limited due to light penetration and regulatory difficulties. Imaging techniques such as EPR and Positron Emission Tomography have shown early potential, but their development for ROS quantification is still in its early stages [152, 153]. Future efforts should focus on developing more advanced tools and techniques for improved *in vivo* ROS detection and monitoring.

An ideal ROS probe—that is also suitable for hypoxia studies—would combine high specificity for individual ROS species, fast and preferably reversible responses, and precise subcellular targeting. It should detect physiological ROS levels with minimal background, provide quantitative or ratiometric readouts, and avoid disrupting redox balance or causing toxicity. Ideally, it would also enable multiplexing with other probes and remain stable and effective in live‐cell and *in vivo* settings—features that current probes only partially achieve. In current practice, however, most available probes represent a trade‐off between sensitivity, specificity and feasibility. Fluorescent dyes remain accessible but are prone to artefacts. Genetically encoded sensors provide spatial and temporal resolution but require genetic manipulation and specialised imaging. Oxidative damage biomarkers capture cumulative stress but fail to resolve dynamic fluctuations. EPR and other physical detection methods add specificity but lack adaptability to complex biological systems.

When applied to hypoxic contexts, these limitations become even more pronounced. Low oxygen tensions can directly interfere with probe chemistry, alter the redox status of cells and introduce reoxygenation artefacts during measurement. Consequently, results obtained with different approaches are often difficult to reconcile, not only due to biological inconsistency but also because each method interrogates a distinct layer of redox biology, such as acute ROS fluxes, compartmental localisation, or downstream damage signatures. For example, fluorescent probes may detect transient ROS bursts missed by oxidative damage assays, whereas biomarker measurements may capture persistent stress in conditions where acute probes show no change.

Thus, the key challenge is not simply to refine existing probes in isolation but to integrate complementary methods into experimental designs that account for temporal dynamics, spatial heterogeneity and repair processes. A multi‐modal approach, for instance combining genetically encoded sensors for live‐cell dynamics with LC–MS‐based biomarker quantification, is likely to provide a more accurate and holistic picture of ROS behaviour under hypoxia. Looking forward, methodological progress will depend on the development of probes that can operate reliably in fluctuating oxygen environments, as well as the adoption of standardised calibration protocols to enable meaningful cross‐study comparisons. Without these advances, conclusions about ROS dynamics in hypoxia will remain highly dependent on the methodological lens through which they are viewed, limiting the field's ability to define consensus mechanisms.

## 6 How can we target ROS effectively in the hypoxic TME?

**Fig. 3:**
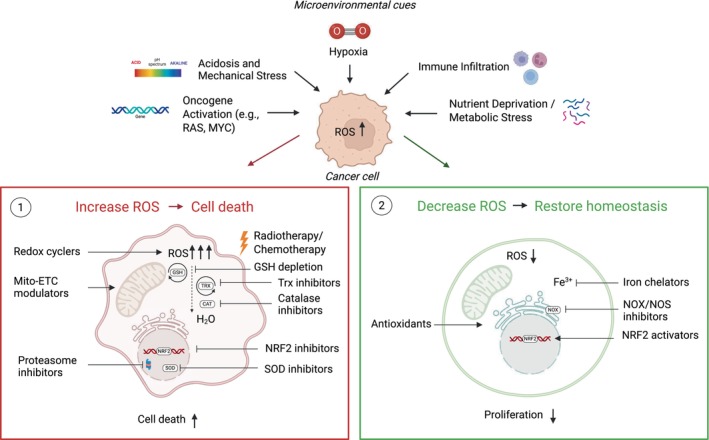
Therapeutic targeting of redox homeostasis in the hypoxic tumour microenvironment. Microenvironmental cues such as hypoxia, acidosis, nutrient deprivation, inflammation and oncogene activation promote ROS accumulation in cancer cells. Two therapeutic strategies exploit this redox imbalance: (1) Increasing ROS to induce cell death; and (2) decreasing ROS to restore cellular homeostasis and reduce proliferation. Created with Biorender.

Modulating ROS levels in the hypoxic TME has been explored as a therapeutic strategy to enhance cancer treatment (Fig. 3) [9]. Many therapeutic approaches take advantage of the fact that cancer cells maintain elevated ROS levels, aiming to overwhelm their redox adaptation mechanisms and induce oxidative stress that is incompatible with cellular survival. Conversely, some strategies aim to reduce ROS levels to inhibit cancer progression. Both approaches target either ROS generation machinery directly or disrupt redox adaptation mechanisms [154]. Given the ability of ROS to diffuse across cellular membranes [155], achieving the right balance is critical to minimise toxicity to normal cells. Notably, testing therapeutics under physiologically relevant O_2_ conditions is essential, as the low O_2_ environment in the TME can significantly impact ROS levels and drug efficacy [156].

### 6.1 Enhancing ROS to induce tumour cell death

ROS elevation strategies either focus on increasing ROS production directly or on weakening the antioxidant defences that maintain ROS balance. For example, the tyrosine kinase inhibitor Anlotinib induces ROS production directly through NOX5 upregulation, leading to apoptosis in squamous cell carcinoma [157]. Strategies that weaken antioxidant defences usually target key components of the defence machinery, such as NRF2, glutathione, and thioredoxin. NRF2 has been effectively inhibited by ML385 and clobetasol propionate to enhance radiotherapy effects under hypoxia [158, 159, 160, 161, 162, 163]. The glutathione synthesis inhibitor buthionine sulfoxide increases ROS but is less effective in hypoxia due to compensatory pathways [164, 165]. The glutaminase inhibitor CB‐839 (Telaglenastat), which limits glutamine‐derived glutathione synthesis, has also been shown to induce ROS and enhance radiation‐induced tumour cell death [166, 167, 168]; however, its effects under hypoxia remain to be determined. Inhibiting thioredoxin reductase with Auranofin enhances ROS accumulation and disrupts hypoxia‐related pathways, such as reducing downstream vascular endothelial growth factor expression [169, 170]. Direct intratumoural injection of H_2_O_2_ in combination with radiotherapy has been explored to enhance cytotoxicity through increased DNA damage and lysosomal membrane permeabilisation, as well as to improve tumour oxygenation, thereby increasing radiosensitivity [171, 172].

FDA‐approved drugs including cisplatin, arsenic trioxide, doxorubicin and paclitaxel modulate ROS levels [4]. For example, doxorubicin, a well‐known topoisomerase II inhibitor and ROS inducer in cancer cells, is effective in various types of cancer, but its efficacy is impaired in hypoxia (1% O_2_) [173, 174]. Similarly, two studies have indicated that the efficacy of As_2_O_3_, which generates ROS via disruption of the electron transport chain (ETC), is diminished in hypoxia (>1%O_2_) [175, 176]. Several repurposed drugs also act through ROS modulation. Atovaquone, an antimalarial agent, reduces mitochondrial oxygen consumption, thereby increasing ROS levels and sensitising tumours to radiation [177, 178, 179]. Papaverine, a vasodilator, enhances ROS production and has demonstrated potential for improving radiosensitivity [180]. ROS‐inducing nanoparticles are also emerging as a tool for cancer therapy, improving drug delivery and bioavailability. Ferric complexes combined with catalase and CaO_2_ increase ROS while alleviating hypoxia, promoting apoptosis [181], while silver clusters (Ag5) target cysteine oxidation in glutathione and thioredoxin, leading to ROS accumulation and selective tumour cell death, but also with reduced efficacy under hypoxic conditions [42, 182].

### 6.2 Reducing ROS to prevent tumour progression

Some therapeutic approaches follow the strategy of reducing ROS levels to counteract their role in cellular transformation. Iron chelators such as Deferasirox suppress ROS levels and induce apoptosis in cancer cells [183]. Inhibitors of NOX enzymes, such as GKT831, lower ROS generation and sensitise tumour cells to radiation [184, 185]. Inhibition of the nitric oxide synthase (NOS) has been demonstrated to reduce cytokine production and suppress the invasive phenotype of oral cancer cells in 3D culture models [186]. Study results of direct treatments with antioxidants have been mixed [187]. Some, including vitamin E, have been linked to increased tumour growth and metastasis, while high‐dose vitamin C has shown selective cytotoxicity in KRAS (Kirsten rat sarcoma viral oncogene homologue) and BRAF (B‐Raf proto‐oncogene, serine/threonine kinase) mutant colon cancer cells [188].

In conclusion, therapeutic strategies aimed at modulating ROS levels in the hypoxic TME show promise in sensitising tumours to treatment. Importantly, many existing cancer therapies are already thought to affect ROS levels, although often through mechanisms that are poorly understood or not yet fully described. What emerges from current evidence is that the success of ROS‐targeting approaches depends not only on whether ROS are increased or decreased, but on how precisely they intersect with the metabolic and redox landscape of hypoxic cancer cells. Because hypoxia alters both ROS generation (e.g. via ETC activity, NOX upregulation, or fluctuating oxygen availability) and antioxidant defences (e.g. NRF2, glutathione, thioredoxin), interventions may have opposite effects depending on the oxygen context. Drugs that strongly induce ROS in normoxia may lose potency in hypoxia, while others that appear ineffective *in vitro* may gain selectivity in the fluctuating oxygen tensions of an *in vivo* tumour.

A critical challenge is therefore to align therapeutic design with the dynamic and compartmentalised nature of ROS in the hypoxic TME. For example, strategies that broadly elevate ROS risk systemic toxicity, whereas approaches that selectively target mitochondrial ROS, NOX‐derived ROS, or antioxidant pathways specific to cancer cells hold greater potential for therapeutic windows. Similarly, the distinction between chronic and intermittent hypoxia is crucial as intermittent hypoxia tends to generate higher and more damaging ROS bursts [94], suggesting that therapies exploiting this dynamic may differ fundamentally from those designed for steady‐state hypoxia.

Moving forward, effective translation will require careful tumour‐specific profiling of ROS sources, levels and adaptive pathways, combined with *in vivo* models that capture the spatial and temporal heterogeneity of the TME. The development of redox‐based biomarkers to stratify patients and monitor treatment responses will also be essential. Only by integrating mechanistic insights with precise patient‐tailored strategies can ROS modulation be transformed from a broadly conceptual approach into a clinically actionable therapeutic framework.

## 7 Conclusion and perspectives

The varied findings on ROS in tumour hypoxia highlight the complexity of both ROS biology and hypoxic environments. ROS detection is inherently challenging due to their short lifespan, low steady‐state concentrations and dynamic fluctuations in O_2_ levels. Addressing these challenges requires precise identification of ROS species, a clear understanding of measurement techniques, and physiologically relevant experimental conditions.

Developing accurate methods for ROS detection in hypoxia, starting *in vitro* and advancing to *in vivo* models, must be a priority. Measurement tools should be highly selective and capable of withstanding the challenges of live tissue environments, ideally providing spatiotemporal data in fluctuating O_2_ conditions. Achieving this may require the continued advancement of genetically encoded sensors with enhanced sensitivity and specificity, as well as innovative probes capable of capturing the real‐time dynamics of specific ROS species within defined subcellular compartments. Encouraging progress has been made with the development of sensors tailored to these demands [184, 189], but further innovations are essential to improve the accuracy, resolution and reliability of ROS measurements in physiologically relevant conditions.

Experimental conditions play a crucial role in obtaining reliable results. Many *in vitro* studies are conducted at 21% O_2_, far from the physiological norm of ~5% O_2_, which can affect the interpretation of ROS dynamics. Ensuring that experiments reflect pericellular O_2_ levels is essential, and the use of hypoxic chambers instead of incubators can help prevent reoxygenation artefacts. Additionally, timely sample fixation is critical, as exposure to ambient conditions can alter ROS levels, leading to inaccurate conclusions.

For advancing ROS research in hypoxia, the precise reporting of experimental conditions is essential. Without clear communication of which ROS species are measured, their subcellular localisation, careful description of the detection methods used, the oxygen levels applied, the duration of exposure, and whether reoxygenation occurs, experimental variability will continue to hinder progress. Greater clarity and consistency in reporting ROS species, detection techniques and oxygenation parameters are critical for enhancing reproducibility and strengthening the translational relevance of hypoxia‐related ROS research.

Ultimately, hypoxia and oxidative stress are central to tumour progression and therapy resistance. Advancing ROS detection, refining experimental models and developing targeted redox‐modulating therapies will provide deeper insights into the mechanisms driving cancer cell survival, proliferation and resistance to treatment. While ROS‐based therapies hold promise, further research is needed to optimise their efficacy, minimise off‐target effects on normal tissues and integrate them seamlessly with existing treatment modalities. Future advancements in imaging technologies and biomarker development could play a crucial role in this process. Addressing these challenges is essential for improving our understanding of tumour biology and enhancing therapeutic strategies.

## Conflict of interest

The authors declare no competing interests.

## Author contributions

LH conducted the literature search, compiled data and drafted the initial manuscript. ES contributed to the literature search. SJC provided subject‐matter expertise and reviewed and edited the manuscript. EMH supervised the project and revised the manuscript. All authors reviewed and approved the final version of the manuscript.
